# Ecotone shifts in southern Madagascar: first barcoding data and six new species of the endemic millipede genus *Riotintobolus* (Spirobolida, Pachybolidae)

**DOI:** 10.3897/zookeys.953.53977

**Published:** 2020-07-27

**Authors:** Thomas Wesener

**Affiliations:** 1 Zoological Research Museum Alexander Koenig (ZFMK), Leibniz Institute for Animal Biodiversity, Adenauerallee 160, D-53113, Bonn, Germany Zoological Research Museum Alexander Koenig Bonn Germany

**Keywords:** biodiversity, Diplopoda, DNA barcoding, littoral rainforest, soil arthropod, spiny forest

## Abstract

Six new species of the Spirobolida millipede genus *Riotintobolus* Wesener, 2009, are described from the spiny forest in southern Madagascar utilising genetic barcoding, drawings and scanning electron microscopy: *Riotintobolus tsimelahy***sp. nov.**, *R. mangatsiaka***sp. nov.**, *R. lavanono***sp. nov.**, *R. bovinus***sp. nov.**, *R. antafoky***sp. nov.** and *R. makayi***sp. nov.** One other *Riotintobolus* population from the spiny forest might represent an additional species based on genetic data, but it cannot be described as no male specimens were collected. At present, the genus *Riotintobolus* Wesener, 2009 has eight species from the spiny forest and two species from the littoral rainforest. A determination key to all ten species of the genus is provided. Molecular data reveal that the two critically endangered species from the humid littoral rainforest are not closely related to one another, but have their closest relative in the dry spiny forest ecosystem. *Riotintobolus mandenensis* Wesener, 2009, only known from the southern littoral rainforest of Mandena is related to *R. tsimelahy***sp. nov.** from the nearby spiny forest at Tsimelahy with a *p*-distance of 11%, while *R. minutus* Wesener, 2009 from the littoral forest of Sainte Luce is more distant to all other *Riotintobolus* species, but more closely related to *R. bovinus***sp. nov.** from the southwestern forest of the Makay.

## Introduction

Madagascar is one of the ten hottest biodiversity hotspots (Myers et al 2000). The long isolation of Madagascar from other land masses (Krause 2003; Ali and Aitchison 2008) makes the island a great model to study the speciation mechanisms in numerous of its endemic animal groups (Vences et al. 2009). The evolutionary mechanisms among the rise of such a stunning diversity of a high number of animal groups on Madagascar are still little understood, as most studies focus on the vertebrate fauna of the island (e.g. Pearson and Raxworthy 2009). Invertebrates might be better suited for studying biogeographical patterns, as they can survive in smaller habitat fragments during climatically unsuitable times (Brühl 1997; Uys et al. 2009; Yeates et al. 2009).

Among the endemic mega-invertebrate fauna on Madagascar are the so-called fire millipedes of the order Spirobolida with their striking red/black coloration (Wesener et al. 2009a, b, 2011a), and the large giant pill-millipedes of the order Sphaerotheriida with stridulation organs in both sexes (Wesener and VandenSpiegel 2009; Wesener et al. 2011b), reaching the size of a small orange when rolled-up (Enghoff 2003). For both groups, numerous species and genera could recently be described from Madagascar (Wesener et al. 2008, 2011a, 2014; Wesener 2009a, b, 2011; Wesener and Enghoff 2009). Especially diverse are the Spirobolida millipedes on Madagascar; previously only known from two genera, 14 new genera could be described since 2008, with numerous species being microendemic and even listed on the IUCN Red List as critically endangered (e.g. Rudolf and Wesener 2017a, b). One of these genera is *Riotintobolus* Wesener, 2009, aptly named after the mining company Rio Tinto, which is currently active in the only known southern littoral rainforests (Vincelette et al. 2007) where two of the critically endangered species of the genus live (Rudolf and Wesener 2017c, d). The other two species of the genus are known from the desert-like spiny forest ecosystem (Du Puy and Moat 1996; Moat and Smith 2007) in southern Madagascar and seem to have a more widespread distribution. *Riotintobolus* is therefore one of the few genera of Malagasy Spirobolida whose species, distributed in littoral rainforests and dry spiny forests, underwent one or several so-called ecotone shifts (Wirta et al. 2008; Wesener et al. 2011a).

An expedition to Madagascar conducted by TW in 2007, as well as sorting through different museum collections, led to the discovery of six additional *Riotintobolus* species, all from the spiny and gallery forests in the desert-like south of Madagascar.

## Material and methods

### Abbreviations

**CAS**California Academy of Sciences, San Francisco, U.S.A;

**CASENT** Entomology collection, California Academia of Sciences;

**FMNH** Field Museum, Chicago, U.S.A;

**FMMC** Insect and Myriapoda collection voucher numbers;

**MZUF**Museum “La Specola”, Florence, Italy;

**SEM** scanning electron microscopy;

**ZFMK**Zoological Research Museum A. Koenig, Leibniz Institute for Animal Biodiversity, Bonn, Germany;

**ZFMK**-**MYR** collection number of the Myriapoda collection at the ZFMK.

### Illustrations

Dissecting and camera lucida drawings were done under an Olympus SZX12 stereo-microscope. For scanning electron microscopy, the samples were dehydrated via an ethanol chain, mounted on stubs and dried overnight. The stub was sputter-coated with 100 nm of gold in a Hummer VI (Anatech, USA) sputtering system. Images were obtained using a Hitachi S-2460 SEM. Multi-layer photographs were taken with a Leica Z6 Imaging-System based at the ZFMK. Stacked images were put together using the software Auto-Montage (Syncroscopy). All images were later modified using Adobe Photoshop version CS2 and assembled into plates using Adobe Illustrator version CS2.

### DNA extraction and sequencing

DNA was extracted from 14 specimens (see Table 1) of *Riotintobolus*: ten of them preserved in 95% ethanol, the remaining in 75% ethanol. The HCO/LCO primer pair (Folmer et al. 1994) was used to sequence a 652 bp fragment of the mitochondrial cytochrome *c* oxidase subunit I (COI) gene. DNA extraction, PCR, purification, and sequencing protocols were identical to those used in a previous study (Wesener et al. 2010). While the COI gene, being a mitochondrial gene as well as containing little resolution at deeper evolutionary splits, is limited in the resolution of a reconstructed phylogeny of the *Riotintobolus* species, we aimed at finding a unique identifier allowing us to study and illustrate the genetic distances between the different species of the genus. All sequences obtained were checked via Blast searches (Altschul et al. 1997), no contaminations were discovered. The sequences were aligned by hand in BioEdit (Hall 1999) together with those obtained during the only other molecular study on Malagasy Spirobolida (Wesener et al. 2011a), using as outgroup taxa specimens of the genera *Spiromimus* DeSaussure & Zehntner, 1901 and *Aphistogoniulus* Silvestri, 1897 as the near outgroup, and a sequence of the species *Madabolus maximus* Wesener & Enghoff, 2008 of the tribe Pachybolini as the far outgroup. All newly sequenced *Riotintobolus* sequences were uploaded to GenBank (Accession #: MT603148–MT603161, see Table 1).

**Table 1.** Specimens sequenced for the Barcoding analysis. GenBank numbers, voucher numbers, and species identification with a shortened location information.

GenBank #	Voucher #	Species
HQ891241.1		*Madabolus maximus* Wesener, 2008
HQ891229.1		*Aphistogoniulus infernalis* Wesener, 2009
HQ891238.1		*Aphistogoniulus vampyrus* Wesener, 2009
HQ891233.1		*Aphistogoniulus sanguineus* Wesener, 2009
HQ891244.1		*Spiromimus triaureus* Wesener & Enghoff, 2009
MT603148	FMNH-INS	*Riotintobolus mandenensis* A, Mandena
MT603149	FMNH-INS	*Riotintobolus mandenensis* B, Mandena
MT603150	ZFMKMYR 9906	*Riotintobolus minutus* A, Sainte Luce S9
MT603151	ZFMY MYR 9907	*Riotintobolus minutus* B, Sainte Luce S9
MT603152	CASENT 9032805	*Riotintobolus aridus*, MGF059
MT603153	ZFMKMYR 9940	*Riotintobolus tsimelahy* A sp. nov., Tsimelahy
MT603154	ZFMKMYR 941	*Riotintobolus tsimelahy* B sp. nov., Tsimelahy
MT603155	CASENT9032808	*Riotintobolus tsimelahy* C sp. nov., Mahavelo
MT603156	ZFMKMYR 9801	*Riotintobolus mangatsia*ka A sp. nov. Mangatsiaka
MT603157	ZFMKMYR 938	*Riotintobolus mangatsiaka* B sp. nov. Mangatsiaka
MT603158	ZFMKMYR 942	*Riotintobolus lavanono* sp. nov., Lavanono
MT603159	ZFMKMYR 939	*Riotintobolus makayi* sp. nov., Makay
MT603160	ZFMKMYR 940	*Riotintobolus bovinus* sp. nov., Makay
MT603161	ZFMKMYR 2438	*Riotintobolus* sp. 01, Faux Cap iv

### DNA analysis

**Figure 1.**
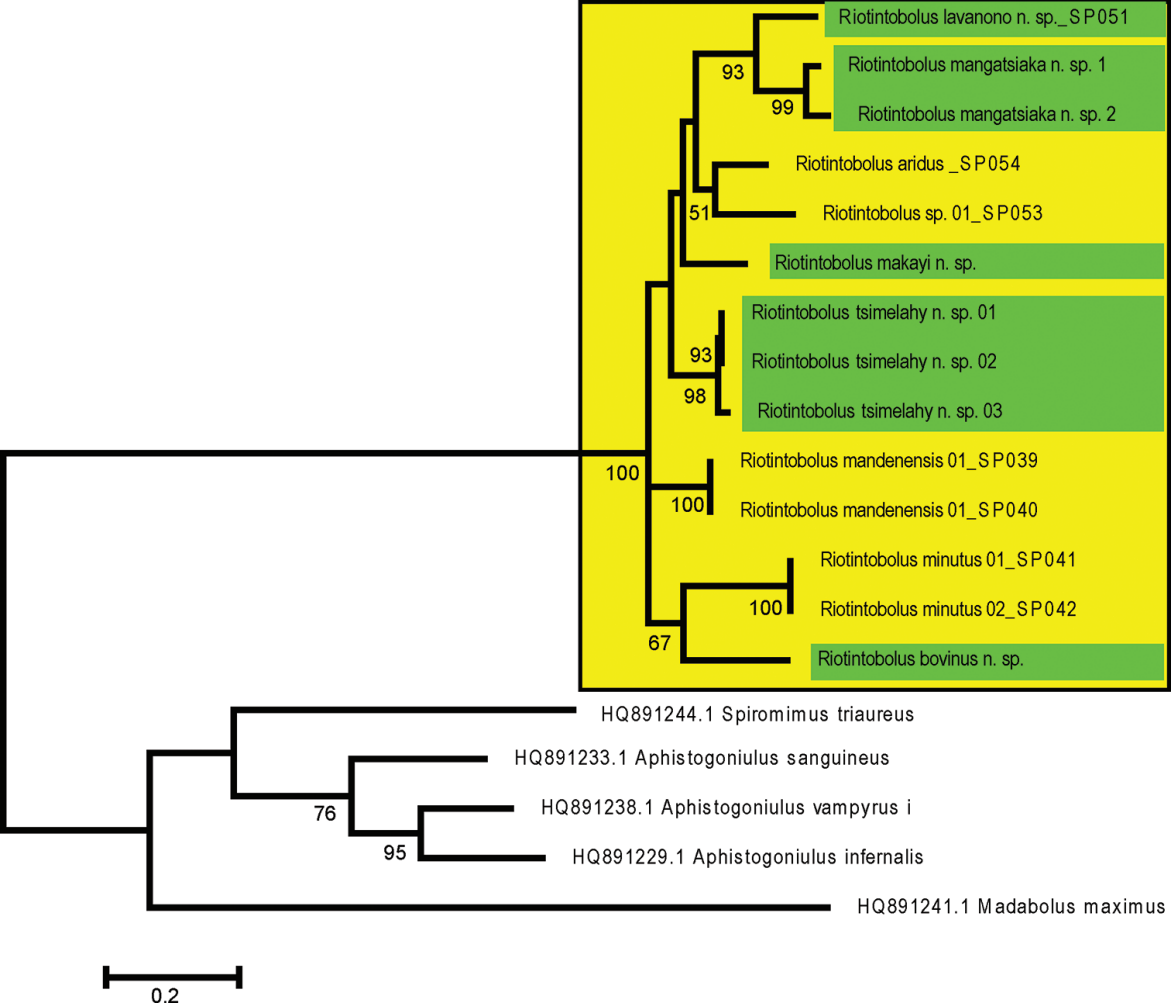
Relationships of *Riotintobolus* species. The evolutionary history of *Riotintobolus* was inferred by using the Maximum Likelihood method based on the Tamura-Nei model. The tree with the highest log likelihood (-5174.8354) is shown. The tree is drawn to scale, with branch lengths measured in the number of substitutions per site. Yellow box marks the genus *Riotintobolus*, green colour bars mark the new species described here. For specimen information, see Table 1. Numbers refer to bootstrap values, values < 50% not shown.

To find the best substitution model, modeltest implemented in MEGA 6 (Tamura et al. 2013) was utilised. Codon positions included were 1st+2nd+3rd. All positions containing gaps and missing data were eliminated. There was a total of 652 positions in the final dataset. The lowest Bayesian Information Criterion score of 10760 was obtained by the Tamura-Nei model plus gamma distribution to be best fitting (FreqA = 0.2848, FreqC = 0.1882, FreqT = 0.3572, FreqG = 0.17, gamma shape = 0.4526). Maximum Likelihood analyses were conducted in MEGA6 (Tamura et al. 2013). The bootstrap consensus tree (Fig. 1) from 1000 replicates (Felsenstein 1985) is taken to represent the evolutionary history of the analysed taxa. The tree with the highest log likelihood (-5174.8354) is shown. Initial tree(s) for the heuristic search were obtained automatically by applying Neighbour-Joining and BioNJ algorithms to a matrix of pairwise distances estimated using the Maximum Composite Likelihood (MCL) approach, and then selecting the topology with superior log likelihood value. A discrete Gamma distribution was used to model evolutionary rate differences among sites (5 categories (+G, parameter = 0.4535)). The tree is drawn to scale, with branch lengths measured in the number of substitutions per site. The analysis involved 19 nucleotide sequences. Codon positions included were 1st+2nd+3rd. All positions with less than 5% site coverage were eliminated. That is, fewer than 95% alignment gaps, missing data, and ambiguous bases were allowed at any position. There was a total of 652 positions in the final dataset. Evolutionary analyses were conducted in MEGA 6 (Tamura et al. 2013). Genetic distances were also analysed in MEGA 6. The analysis involved 19 nucleotide sequences. Codon positions included were 1st+2nd+3rd. All ambiguous positions were removed for each sequence pair. Results are shown in the supplemental material (Suppl. material 1).

## Results

### Genetic distance analyses

Interspecific distances vary inside *Riotintobolus* between 11–16.4%, while intraspecific distances are between 1.5–4.1% (Suppl. material 1). The maximum likelihood analysis of the COI barcoding gene strongly supports the monophyly of *Riotintobolus* (Fig. 1, 100% bootstrap support). Inside *Riotintobolus*, groups of the eight species and the one candidate species are not well-supported, with the exception of *R. mangatsiaka* sp. nov. being related to *R. lavanono* sp. nov. (Fig. 1, 93% bootstrap support). The species, however, are all recovered as monophyletic receiving a bootstrap support of > 90%.

## Taxonomy

### Order Spirobolida, family Pachybolidae sensu Hoffman 1980

#### Genus Riotintobolus

Taxon classificationAnimaliaSpirobolidaPachybolidae

Wesener, 2009

78822015-5DEB-53ED-AB4F-9AD5F37B5BC5

##### Type species.

*Riotintobolus mandenensis* Wesener, 2009, by original designation.

##### Other species included.

*Riotintobolus minutus* Wesener, 2009

*Riotintobolus aridus* Wesener, 2009

*Riotintobolus anomalus* Wesener, 2009

*Riotintobolus tsimelahy* sp. nov.

*Riotintobolus mangatsiaka* sp. nov.

*Riotintobolus lavanono* sp. nov.

*Riotintobolus bovinus* sp. nov.

*Riotintobolus antafoky* sp. nov.

*Riotintobolus makayi* sp. nov.

##### Distribution.

**Figure 2.**
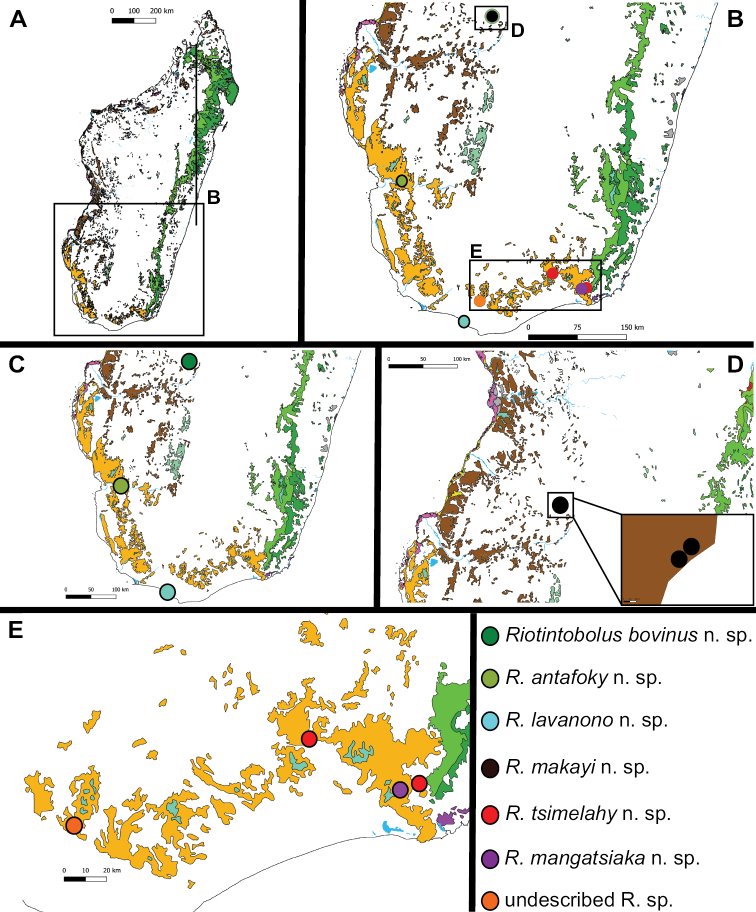
Distribution of the newly described *Riotintobolus* species, as well as of the still undescribed species. Vegetation marks modified from Moat and Smith 2007.

Southern spiny forests and gallery forests, southeastern littoral rainforests (Fig. 2).

### Determination key to the species of *Riotintobolus*

**Table:** 

1	Epiproct of telson projecting into a short process	**2**
–	Epiproct of telson not projecting	**5**
2	Male legs without tarsal pads. Eyes consisting of 20 or less ommatidia. Anal valves with a deep groove between anterior half and sharp-edged lips	**3**
–	Male legs 3–7 with tarsal pads. Eyes with more than 25 ommatidia. Anal valves with neither a groove, nor sharp-edged lips	**4**
3	Specimens 35–43 mm long. Colour laterally black, dorsally with a thick, light brown stripe. Telopodite of posterior gonopod laterally with one large and second small finger-shaped process	***R. mandenensis* Wesener, 2009**
–	Specimens shorter than 25 mm. Colour laterally black, dorsally with a thick, red stripe. Telopodite of posterior gonopod laterally with two large finger-shaped processes of equal size	***R. minutus* Wesener, 2009**
4	Apical membrane on posterior gonopod weakly developed. Telopodite at mesal margin with a conspicuous horn. Process ‘x’ on lateral margin without recurved tip	***R. aridus* Wesener, 2009**
–	Apical membrane on posterior gonopod present as a sail (Fig. 10G: ‘z’). Mesal margin without a horn. Process ‘x’ on lateral margin with a recurved tip bending mesally (Fig. 10G). Efferent duct ending free	***R. makayi* sp. nov.**
5	Posterior telopod, telopodite with two or more slender, sharp projections (Fig. 3E)	**6**
–	Posterior gonopod, telopodite, projecting in a simple ‘flag’ (Fig. 9C)	**9**
6	Males > 40 mm, > 45 segments plus telson. Posterior gonopod separated into three parts (Fig. 3E)	**7**
–	Male ca. 25 mm long, 41 segments plus telson. Posterior gonopod only separated into coxite and telopodite (Fig. 8F)	***R. bovinus* sp. nov.**
7	Antennae and legs red (Fig. 3A). Posterior gonopod, lateral process slender. Membranous mesal area spanning whole mesal side of tip (Fig. 3E: ‘z’)	**8**
–	Antennae and legs dark grey (FIg. 7A). Posterior gonopod, lateral process wide, straight, well-rounded (Fig. 7E). Membranous mesal area globular (Fig. 7E: ‘z’)	***R. lavanono* sp. nov.**
8	Posterior gonopod with two lateral processes (Fig. 3E)	***R. tsimelahy* sp. nov.**
–	Posterior gonopod with single lateral process (Fig. 4I)	***R. mangatsiaka* sp. nov.**
9	Males > 45 mm long, dorsally with a red stripe. Antenna short, protruding back to segment 3. Male legs 3 to at least midbody legs with tarsal pad. Anterior gonopod, coxal process inconspicuous, not reverted. Posterior gonopod, apical part with membranous flag	***R. anomalus* Wesener, 2009**
–	Males only 33 mm long, dorsally without any stripe. Antenna long, protruding back to segment 5. Male legs without a tarsal pad. Anterior gonopod, coxite process, apical margin reverted (Fig. 9A). Posterior gonopod, apical part of telopodite, flag with a sclerotised projection (Fig. 9C)	***R. antafoky* sp. nov.**

#### Riotintobolus tsimelahy sp. nov.

Taxon classificationAnimaliaSpirobolidaPachybolidae

B286BFDB-5D4F-5709-B5DE-F0A154F890A2

http://zoobank.org/672376C7-CC91-4259-B896-B45229104743

Figure 3

##### Material examined.

1 ♂ ***holotype***, **ZFMK MYR9940**, Madagascar, PN Andohahela, Tsimelahy, 24°57.296'S, 046°37.214'E, 135 m, spiny forest, coll. Wesener and Schütte, 24.v.2007.

***Paratypes***: 9 ♂, 12 ♀, **ZFMK MYR941**, same data as holotype; 1 ♂, **ZFMK MYR9950**, same data as holotype; 1 ♂ with damaged gonopods; **ZFMK MYR9949**, same data as holotype.

##### Other material examined.

3 ♂ and ♀, **BLF 5239 (CASENT9032808)**, Madagascar, Toliara, Forêt de Mahavelo, Isantoria River, 24°45'30"S, 46°9'26"E, 110 m, 28.i-1.ii.2002, spiny forest/thicket, EH18 pitfall trap, coll. B. L. Fisher et al.; 1 ♂, 1 imm., **MZUF Fi-10**, Madagascar; Andohahela, 6–12.xii.1991, leg B. Randriamampionona.

##### Etymology.

Tsimelahy, after the type locality (Fig. 2), spiny forests next to the Tsimelahy River, Andohahela National Parc. Noun in apposition.

##### Diagnosis.

*Riotintobolus tsimelahy* sp. nov. shares the absence of a projecting epiproct on the telson only with *R. anomalus*, *R. antafoky* sp. nov., *R. bovinus* sp. nov., *R. mangatsiaka* sp. nov. and *R. lavanono* sp. nov. The posterior telopod featuring two slender, sharp projections is only shared with *R. bovinus* sp. nov., *R. mangatsiaka* sp. nov. and *R. lavanono* sp. nov. A posterior gonopod separated into three parts is only shared with *R. mangatsiaka* sp. nov. and *R. lavanono* sp. nov., whose habitus and gonopods look very similar to those of *R. tsimelahy* sp. nov. Both species differ in details of the tip of the posterior gonopod and in the colour of their antennae and legs, which are red in *R. tsimelahy* sp. nov. and dark grey in *R. lavanono* sp. nov. *R. tsimelahy* sp. nov. differs from *R. mangatsiaka* sp. nov. in the presence of two lateral processes on the posterior gonopod. All three species differ by >11% uncorrected p-distance in the COI barcoding gene.

##### Description.

***Measurements***: Telson not included in counts of segments. Male holotype with 50+0 segments, ca. 44 mm long, 4.3 mm wide. Largest females of type series with 49 or 50+0 segments, up to 50 mm long, 5.3 mm wide.

**Figure 3.**
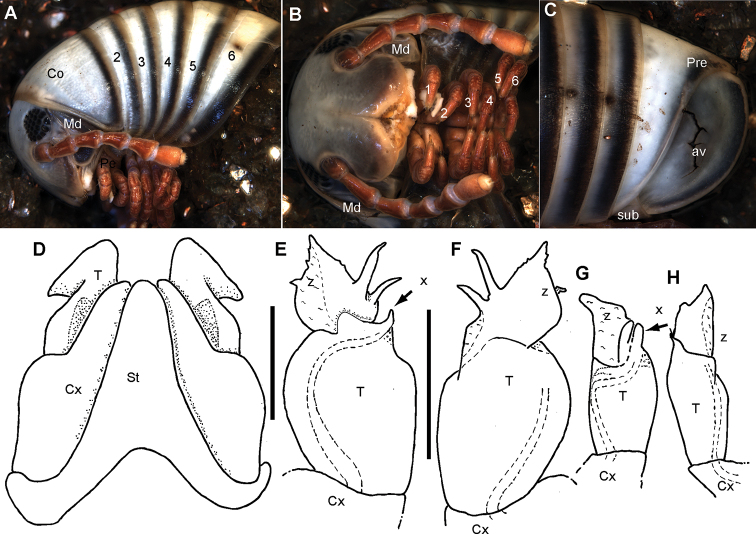
*Riotintobolus tsimelahy* sp. nov., holotype (ZFMK MYR99940) (**A–F**) and male paratype (ZFMK MYR9949) (**G, H**) **A** multi-layer photograph, male anterior body, lateral view **B** multi-layer photograph, male leg pairs 1–7, ventral view **C** multi-layer photograph, male posterior body end with telson **D** anterior gonopod, anterior view **E** left posterior gonopod, posterior view **F** right posterior gonopod, anterior view **G** damaged? left posterior gonopod, posterior view **H** damaged? right posterior gonopod, anterior view. **Abbreviations**: av = paraprocts; Co = collum (ring 1); Cx = coxite; Md = basal joints of mandible; Pre = epiproct; St = sternite; sub = hypoproct; T = telopodite; x = lateral swollen process; z = apical membranous ‘flag’; numbers refer to leg pair number. Scale bars: 1 mm.

***Colour*** (in living specimens): Body rings grey, appendages red. Head, paraprocts and posterior margins of body segments darker grey to black. Ozopore openings highlighted by black spot (Fig. 3A, C).

***Head***: each eye with 30–35 ommatidia in six rows. Incisura lateralis open (Fig. 3A). Labrum with standard three irregular teeth and a single row of 10–12 stout marginal setae. Clypeus with two setiferous foveolae on each side (Fig. 3B). Antennae long, protruding back to segment 5. Length of antennomeres: 1<2>3=4=5=6. Second antennomere slenderer but twice as long as first. Terminal antennomere with four large sensory cones located together inside a membranous area.

***Gnathochilarium***: lamellae linguales each with two standard setae located behind one another. Stipites each with three apical setae. Palpi of similar size. Endochilarium not dissected.

***Mandible*** not dissected.

***Collum***: smooth, laterally not protruding as far as ring 2 (Fig. 3A).

***Body rings***: ozopores starting at segment 6, marked by a black spot. Located on suture between meso- and metazonite. Rings with smooth, but irregular coriaceous surface, ventrally on metazona with transverse ridges.

***Telson***: paraprocts elongated, with weak lips, abundant micropunctation especially towards edges (Fig. 3C). Epiproct well-rounded, covering, but not reaching above paraproct (Fig. 3C). Hypoproct inconspicuous (Fig. 3C).

***Legs***: leg 1 with a large cylindrical coxa, twice as long as other podomeres. Tarsus with three pairs of ventral spines and an apical spine beyond claw. Leg 2 with an elongated coxa. Tarsus with three pairs of ventral spines and a short apical spine. Midbody legs with a rectangular coxa, as long as other podomeres. Each podomere ventrally with a single or a pair of apical setae, tarsus with a single apical and three pairs of ventral spines. Length of midbody legs ca. 1.2 times body diameter in males.

***Female sexual characters*.** No tarsal pads, antennae shorter than male, only protruding back to ring 2. Vulvae not dissected.

***Male sexual characters***: tarsal pads present from leg 3 to midbody, small, inconspicuous (Fig. 3B). Coxae 3–7 without coxal processes, but coxae 3–5 swollen (Fig. 3B).

***Anterior gonopod*** sternite massive (Fig. 3D), elongated into a wide, well-rounded triangular lobe (Fig. 3D). Sternite in anterior view well-visible, without discernible apodemes, protruding almost higher than coxal processes. Coxite with a large, well-rounded mesal process (Fig. 3D). Telopodite with process arising mesally (Fig. 3D), process apically curved with a large triangular projection (Fig. 3D), tip well-rounded, slightly protruding above lateral margin of telopodite (Fig. 3D). Whole telopodite process resembling an even-sided triangle.

***Posterior gonopods*** consisting of three parts, separated by sutures or articulations: a basal coxite with a slender coxite projection and a slightly shorter telopodite, efferent duct discharging laterally (Fig. 3E, F). Process of coxite and telopodite standing in same axis (Fig. 3E, F). Pair of posterior gonopods located parallel to each other, connected by a small, sclerotised and visible sternite. Basal part of coxite wide, mesally with a large triangular sclerite located on lower level than remaining part. Coxite elongated. Efferent duct running at mesal margin of coxite (Fig. 3E) before curving to the lateral margin at beginning of telopodite (Fig. 3E). Telopodite as wide as but much shorter than coxite, standing in same axis (Fig. 3F), apically membranous, with one triangular apical process and two slender lateral processes (Fig. 3E, F). Lateral processes straight, running almost parallel to one another, slender and sclerotised, efferent duct seems to be ending at base of lateral process (Fig. 3E). Base of lateral process with a short, membranous-white projection (Fig. 3E).

##### Intraspecific variation.

The number of segments varies, even within one population, between 47 and 51. The population from the Forêt de Mahavelo (Fig. 1) differs by 1.5% uncorrected p-distance of the COI gene to those from the type locality, Tsimelahy (Suppl. material 1). One small male from the type series (ZFMK MYR9949) has incompletely developed posterior gonopods, either a sign of healed damage (there are black spots on it), or maybe not fully developed (FiIg. 3G, H).

##### Live observations.

*R. tsimelahy* sp. nov. could be found in great numbers in the early morning (7–9 a.m.) on the forest floor of the spiny bush. The otherwise dry spiny bush was still quite wet because of dew. No juveniles were observed. Contrary to other *Riotintobolus* species, such as *R. mandenensis* and *R. minutus*, *R. tsimelahy* sp. nov. did not remain stiff like a stick when disturbed, but rolled-up into a spiral, a common defence behaviour for juliform millipedes.

#### Riotintobolus mangatsiaka sp. nov.

Taxon classificationAnimaliaSpirobolidaPachybolidae

19C86D9F-ED4A-52FF-944A-A3198B4B705B

http://zoobank.org/9B563AE0-D187-481A-893B-88DF1CE920A3

Figures 4 , 5 , 6

##### Material examined.

1 ♂ ***holotype***, **ZFMK MYR9801**, Madagascar, PN Andohahela, Mangatsiaka, 24°58.051'S, 046°33.206'E, 90 m, spiny forest, leg. Wesener and Schütte, 23.v.2007.

***Paratypes***: 7 ♂, 14 ♀, **ZFMK MYR938**, same data as holotype.

##### Other material examined.

1 ♂, 1 ♀, **FMMC 5413**, Province Toliara; RNI d’Andohahela, parcel 2; 120 m; 24°49.0'S, 46°36.6'E; pitfalls, camp 6; leg. S. Goodman 7–15.xii.1995; 1 ♂, **FMMC 5379**, Province Toliara; RNI d’Andohahela, parcel 2; 120 m; 24°49.0'S, 46°36.6'E; pitfalls, camp 6; leg. S. Goodman 7–15.xii.1995;

##### Etymology.

Mangatsiaka, after the type locality (Fig. 2), spiny forests next to a site called Mangatsiaka, Andohahela National Parc. Noun in apposition.

##### Diagnosis.

*Riotintobolus mangatsiaka* sp. nov. shares the absence of a projecting epiproct on the telson only with *R. anomalus*, *R. antafoky* sp. nov., *R. bovinus* sp. nov., *R. tsimelahy* sp. nov. and *R. lavanono* sp. nov. The posterior telopod featuring two slender, sharp projections is only shared with *R. bovinus* sp. nov., *R. tsimelahy* sp. nov. and *R. lavanono* sp. nov. A posterior gonopod separated into three parts is only shared with *R. tsimelahy* sp. nov. and *R. lavanono* sp. nov., whose habitus and gonopods look very similar to those of *R. mangatsiaka* sp. nov. Both species differ in details of the tip of the posterior gonopod and in the colour of their antennae and legs, which are red in *R. mangatsiaka* sp. nov. and dark grey in *R. lavanono* sp. nov. *R. mangatsiaka* sp. nov. differs from *R. tsimelahy* sp. nov. in the presence of just one lateral processes on the posterior gonopod. All three species differ by >11% uncorrected p-distance in the COI barcoding gene.

##### Description.

***Measurements***: male holotype with 49+0 segments, ca. 42 mm long, 4.1 mm wide. Largest females of type series with 48 to 51+0 segments, up to 52 mm long, 5.4 mm wide.

**Figure 4.**
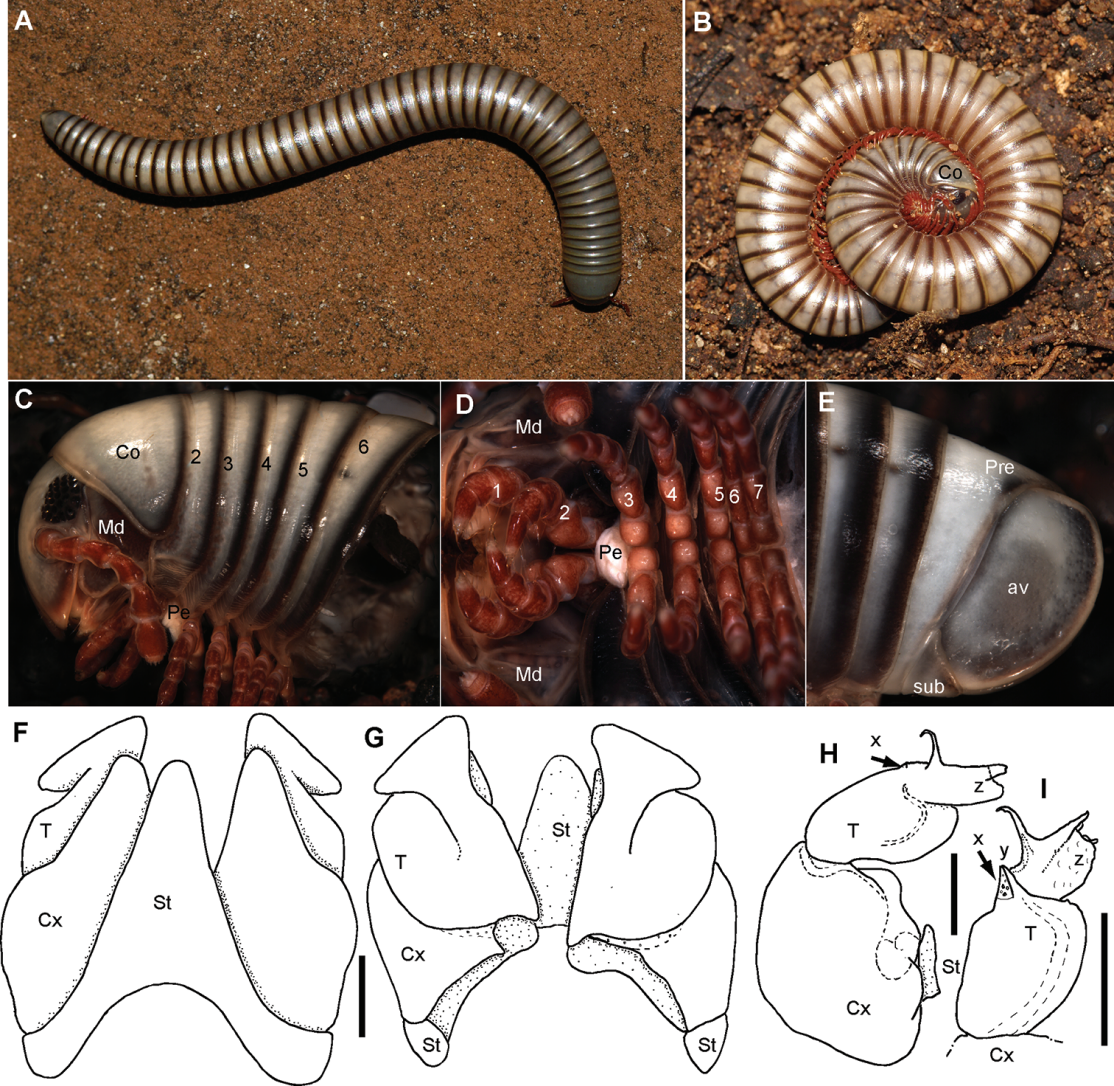
*Riotintobolus mangatsiaka* sp. nov., paratypes (ZFMK MYR938) **A** living female **B** living male in defensive position **C** multi-layer photograph, male anterior body, lateral view **D** multi-layer photograph, male leg pairs 1–7, ventral view **E** multi-layer photograph, male posterior body end with telson **F** anterior gonopod, anterior view **G** anterior gonopod, posterior view **H** right posterior gonopod, posterior view **I** right posterior gonopod, anterior view. **Abbreviations**: av = paraprocts; Co = collum (ring 1); Cx = coxite; Md = basal joints of mandible; Pe = penis; Pre = epiproct; St = sternite; sub = hypoproct; T = telopodite; x = lateral swollen process; y = opening of efferenct duct; z = apical membranous ‘flag’; numbers refer to leg pair number. Scale bars: 1 mm.

***Colour*** (in living specimens): Body rings grey, appendages red. Head, paraprocts and posterior margins of body segments darker grey to black (Fig. 4A). Ozopore openings highlighted by black spot (Fig. 4A, B).

**Figure 5.**
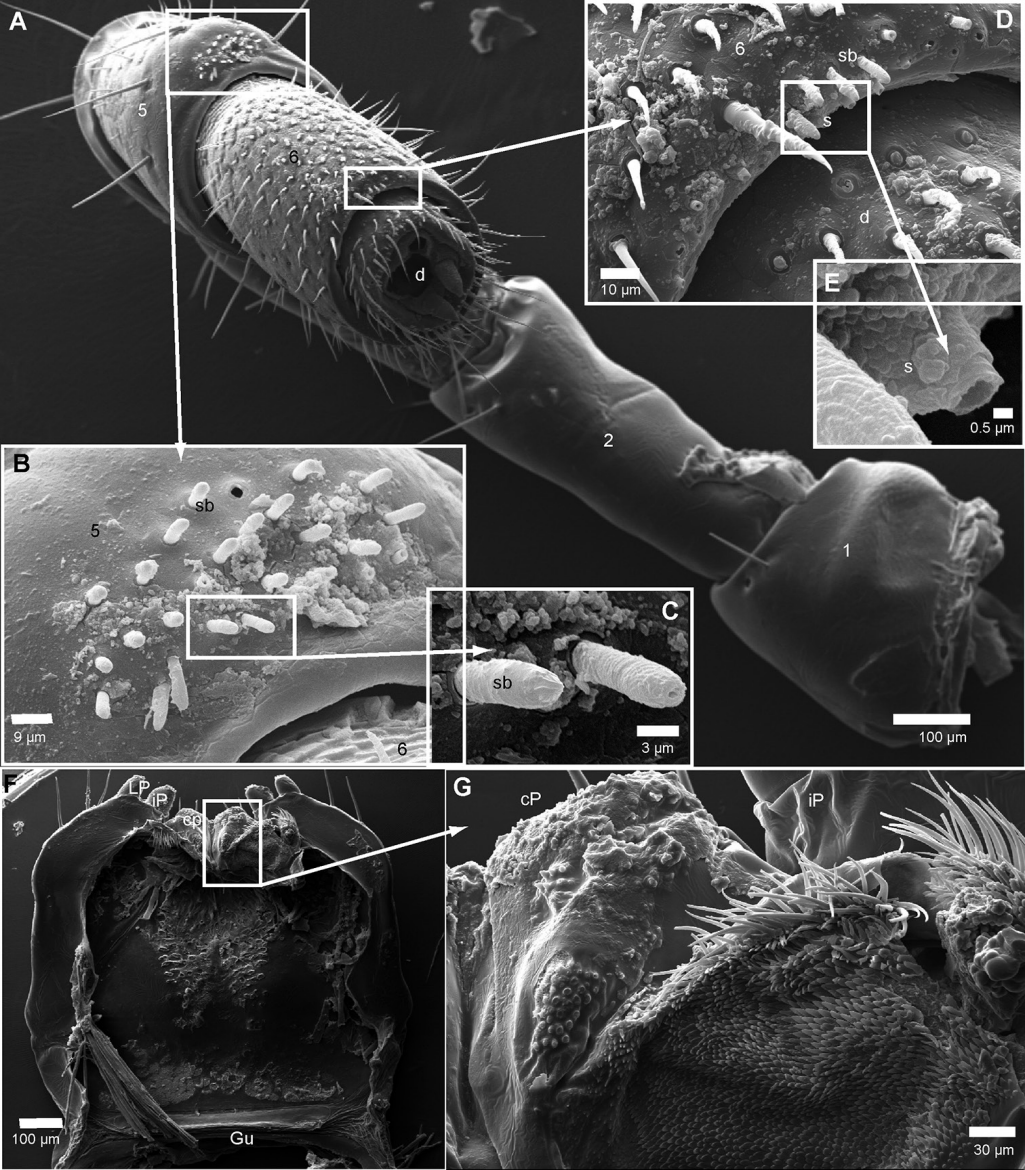
*Riotintobolus mangatsiaka* sp. nov., male (FMMC 5413), SEM**A** left antennae **B** antennomere 5 **C** details of sensilla basiconica **D** antennomere 6 **E** detail of unknown sensilla/pore opening **F** gnathochilarium underside **G** gnathochilarium, detail of right central pad. **Abbreviations**: cP = central pads; d = disc; iP = inner palpus; LP = lateral palpus; s = unknown sensillum; sb = sensilla basiconica; numbers refer to antennomere number. Scale bars as indicated.

***Head***: each eye with 30–35 ommatidia in six rows. Incisura lateralis open (Fig. 4C). Labrum with standard three irregular teeth and a single row of 10–12 stout marginal setae. Clypeus with two setiferous foveolae on each side (Fig. 4D). Antennae long, protruding back to segment 5. Length of antennomeres: 1<2>3=4=5=6. Second antennomere slenderer but twice as long as first. Terminal antennomere with four large sensory cones located together inside a membranous area (Fig. 5A). Antennomere 5 with 3 rows, antennomere 6 latero-apically with a single row of sensilla basiconica (Fig. 5B–E). Antennomere 6 with an unknown type of at least three sensilla or duct openings located close to disc (Fig. 5D, E).

***Gnathochilarium***: lamellae linguales each with two standard setae located behind one another. Stipites each with three apical setae. Palpi of similar size (Fig. 5F). Central pads with standard two sets of sensory cones, apical area with ten cones, higher area with ~ 30 (Fig. 5G).

**Figure 6.**
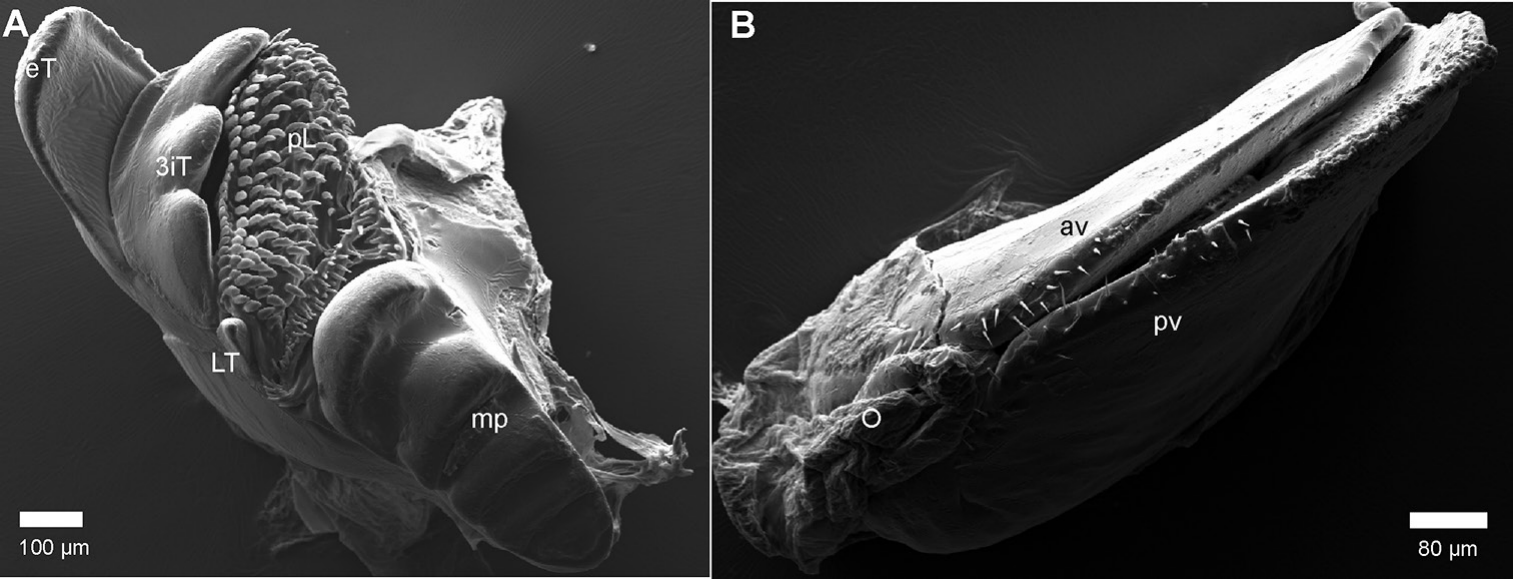
*Riotintobolus mangatsiaka* sp. nov., male and female (FMMC 5413), SEM**A** left gnathal lobe of mandible, mesal view **B** left female vulva, lateral view. **Abbreviations**: av = anterior orientated plate; 3iT = 3-combed inner tooth; eT ? external tooth; LT = lateral tooth; mp = molar plate; O = operculum; pL = pectinate lamellae; pv = posterior orientated plate. Scale bars as indicated.

***Mandible***: Stipes without projection, well rounded (Fig. 4C). Gnathal lobe, external tooth simple, rounded; mesal tooth with three cusps (Fig. 6A). Eight pectinate lamellae. Mesal margin of pectinate area (intermediate area) with ca. four rows of small, slender spines. Molar plate with few, five, transverse furrows (Fig. 6A).

***Collum***: smooth, laterally not protruding as far as ring 2 (Fig. 4C).

***Body rings***: ozopores starting at segment 6, marked by a black spot. Located on suture between meso- and metazonite. Rings with smooth, but irregular coriaecous surface, ventrally on metazona with transverse ridges.

***Telson***: paraprocts elongated, with weak lips, abundant micropunctation especially towards edges (Fig. 4E). Epiproct well-rounded, covering, but not reaching above paraproct (Fig. 4E). Hypoproct inconspicuous (Fig. 4E).

***Legs***: leg 1 with a large cylindrical coxa, twice as long as other podomeres. Tarsus with three pairs of ventral spines and an apical spine beyond claw. Leg 2 with an elongated coxa. Tarsus with three pairs of ventral spines and a short apical spine. Midbody legs with a rectangular coxa, as long as other podomeres. Each podomere ventrally with a single or a pair of apical setae, tarsus with a single apical spine and three pairs of ventral spines. Length of midbody legs ca. 1.2 times body diameter in males.

***Female sexual characters*.** No tarsal pads, antennae shorter than male, only protruding back to ring 2. Female vulva simple, bivalve-like (Fig. 6B). Anterior plate smaller than posterior plate, opening with one row of setae on each plate, close to operculum.

***Male sexual characters***: tarsal pads present from leg 3 to midbody, small, inconspicuous (Fig. 4D). Coxae 3–7 without coxal processes, but coxae 3–5 swollen (Fig. 3D).

***Anterior gonopod*** sternite massive (Fig. 4F), elongated into a wide, well-rounded triangular lobe (Fig. 4F). Sternite in anterior view well-visible, without discernible apodemes, protruding almost as high as coxal processes. Coxite with a large, well-rounded mesal process (FIg. 4F, G). Telopodite with process arising mesally (Fig. 4G), process apically curved with a large triangular projection (Fig. 4G), tip well-rounded, slightly protruding above lateral margin of telopodite (Fig. 4F). Whole telopodite process resembling an even-sided triangle.

***Posterior gonopods*** consisting of three parts, separated by sutures or articulations (Fig. 4H): a basal coxite with a slender coxite projection and a slightly shorter telopodite, efferent duct discharging laterally (FIg. 4H, I). Process of coxite and telopodite standing in same axis (Fig. 4H). Pair of posterior gonopods located parallel to each other, connected by a small, sclerotised and visible sternite (Fig. 4H). Basal part of coxite wide, mesally with a large triangular sclerite located on lower level than remaining part (Fig. 4H). Coxite elongated. Efferent duct running at mesal margin of coxite (FIg. 4H, I) before curving to the lateral margin at beginning of telopodite (Fig. 4I). Telopodite as wide as but much shorter than coxite, standing in same axis (FIg. 4H, I), apically membranous, with two slender apical processes both diverging (FIg. 4H, I). Mesal process membranous and wider, lateral process longer, slenderer and sclerotised, efferent duct seems to be ending at base of lateral process (FIg. 4H, I). Base of lateral process with a short, membranous-white projection (Fig. 4I).

##### Intraspecific variation.

The number of segments varies between 47 and 51.

##### Live observations.

*R. mangatsiaka* sp. nov. could be found in great numbers in the early morning (7–9 a.m.) on the forest floor of the spiny bush. The otherwise dry spiny bush was still quite wet because of dew. No juveniles were observed. Contrary to other *Riotintobolus* species, such as *R. mandenensis* and *R. minutus*, *R. mangatsiaka* sp. nov. did not remain stiff like a stick when disturbed, but rolled-up into a spiral (Fig. 4B), a common defence behaviour for juliform millipedes.

#### Riotintobolus lavanono sp. nov.

Taxon classificationAnimaliaSpirobolidaPachybolidae

38CDFD0A-2BCC-529B-BD39-F39BA0032BFA

http://zoobank.org/4BBC68AE-EAB0-4311-88EA-DDCFD5C1882F

Figure 7

##### Material examined.

1 ♂ ***holotype***, **ZFMK MYR9941**, Madagascar, South, Lavanono Beach, 25°25.404'S, 044°56.414'E, 27 m, spiny bush at the coast, after rain, leg. Wesener and Schütte, 18.vi.2007.

***Paratypes***: 16 ♂, 18 ♀, **ZFMK MYR942**, same data as holotype;

##### Etymology.

Lavanono, after the type locality, spiny forests directly next to the Lavanono Beach (Fig. 2). Noun in apposition.

##### Diagnosis.

*Riotintobolus lavanono* sp. nov. shares the absence of a projecting epiproct on the telson with *R. anomalus*, *R. antafoky* sp. nov., *R. bovinus* sp. nov., *R. tsimelahy* sp. nov. and *R. mangatsiaka* sp. nov., The posterior telopod featuring two slender, sharp projections is only shared with *R. bovinus* sp. nov., *R. mangatsiaka* sp. nov. and *R. tsimelahy* sp. nov. A posterior gonopod separated into three parts is only shared with *R. mangatsiaka* sp. nov. and *R. tsimelahy* sp. nov., whose habitus and gonopods look very similar to those of *R. lavanono* sp. nov. Both species differ in details of the tip of the posterior gonopod and in the colour of their antennae and legs, which are dark grey in *R. lavanono* sp. nov. and red in both *R. mangatsiaka* sp. nov. and *R. tsimelahy* sp. nov. All three species differ by >11% uncorrected p-distance in the COI barcoding gene.

##### Description.

***Measurements***: male holotype with 47+0 segments, ca. 42 mm long, 4.2 mm wide. Largest females of type series with 46–48+0 segments, up to 58 mm long, 6.4 mm wide.

**Figure 7.**
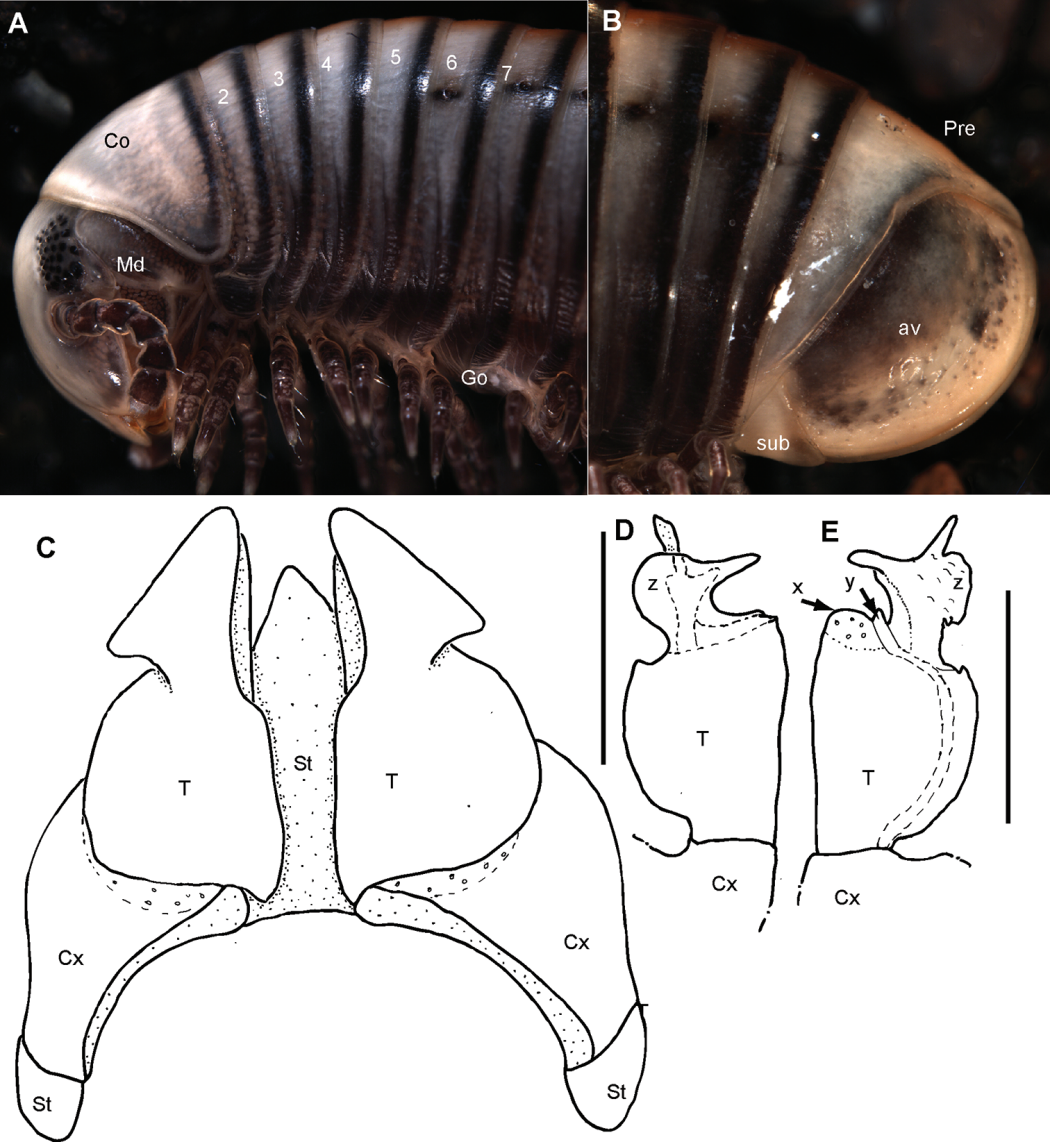
*Riotintobolus lavanono* sp. nov., paratype male (ZFMK MYR942) **C** anterior gonopod, anterior view; posterior view **D** telopodite of left posterior gonopod, posterior view **E** left posterior gonopod, anterior view. **Abbreviations**: av = paraprocts; Co = collum (ring 1); Cx = coxite; Go = gonopods; Md = basal joints of mandible; Pre = epiproct; St = sternite; sub = hypoproct; T = telopodite; x = lateral swollen process; y = opening of efferent duct; z = apical membranous ‘flag’; numbers refer to leg pair or segment number. Scale bars: 1 mm.

***Colour*** (in living specimens): Body rings and head grey, appendages black (Fig. 7A). Paraprocts and posterior margins of body segments darker grey to black (Fig. 7B). Ozopore openings highlighted by black spot.

***Head***: each eye with 28–32 ommatidia in six rows. Incisura lateralis open (Fig. 7A). Labrum with standard three irregular teeth and a single row of 10–12 stout marginal setae. Clypeus with two setiferous foveolae on each side. Antennae long, protruding back to segment 5. Length of antennomeres: 1<2>3>4=5=6. Terminal antennomere with four large sensory cones located together inside a membranous area. Antennomere 5 and 6 latero-apically with sensilla basiconica.

***Gnathochilarium***: lamellae linguales each with two standard setae located behind one another. Stipites each with three apical setae. Endochilarium not dissected.

***Mandible***: Stipes without projection, well rounded (Fig. 7A). Gnathal lobe not investigated.

***Collum***: smooth, laterally not protruding as far as ring 2 (Fig. 7A).

***Body rings***: ozopores starting at segment 6, marked by a black spot. Located on suture between meso- and metazonite. Rings with smooth, but irregular coriaceous surface, ventrally on metazona with transverse ridges.

***Telson***: paraprocts elongated, with weak lips, abundant micropunctation especially towards edges (Fig. 7B). Epiproct well-rounded, covering, but not reaching above paraproct (Fig. 7B). Hypoproct inconspicuous (Fig. 7B).

***Legs***: leg 1 with a large cylindrical coxa, twice as long as other podomeres. Tarsus with three pairs of ventral spines and an apical spine beyond claw. Leg 2 with an elongated coxa. Tarsus with three pairs of ventral spines and a short apical spine. Midbody legs with a rectangular coxa, as long as other podomeres. Each podomere ventrally with a single or a pair of apical setae, tarsus with a single apical and four pairs of ventral spines. Length of midbody legs ca. 1.2 times body diameter in males.

***Female sexual characters*.** No tarsal pads, antennae shorter than male, only protruding back to ring 2. Female vulva simple, bivalve-like.

***Male sexual characters***: tarsal pads present from leg 3 to midbody, small, inconspicuous. Coxae 3–7 without coxal processes, but coxae 3–5 swollen.

***Anterior gonopod*** sternite massive, elongated into a wide, well-rounded triangular lobe (Fig. 7C). Sternite in anterior view well-visible, without discernible apodemes, protruding almost as high as coxal processes. Coxite with a large, well-rounded mesal process. Telopodite with slender process arising mesally (Fig. 7C), process apically curved with a large triangular projection, tip well-rounded, slightly protruding above lateral margin of telopodite.

***Posterior gonopods*** consisting of three parts, separated by sutures or articulations (Fig. 7D): a basal coxite with a slender coxite projection and a shorter telopodite, efferenct duct discharging laterally (Fig. 7E). Process of coxite and telopodite standing in same axis. Pair of posterior gonopods located parallel to each other, connected by a small, sclerotised and visible sternite. Basal part of coxite wide, mesally with a large triangular sclerite located on lower level than remaining part. Coxite elongated. Efferent duct running at mesal margin of coxite before curving to the lateral margin at beginning of telopodite (Fig. 7E). Telopodite half as wide and much shorter than coxite, standing in same axis, apically membranous, with two slender apical processes both diverging (Fig. 7D, E). Mesal process membranous and wider, lateral process bent 90 degrees laterally, longer, slenderer and sclerotised, efferent duct seems to be ending at base of lateral process (Fig. 7E). Base of lateral process with a short, membranous-white projection (Fig. 7E).

##### Intraspecific variation.

Specimens of the same population differing between 45–47 in segment number. Females appear to be more brownish than the more greyish males.

##### Live observations.

*R. lavanono* sp. nov. could be found in great numbers after a rainy day in the late afternoon (3–5 p.m.) in a small remnant of spiny bush and under dead *Opuntia* remains. The specimens were only encountered in an area of a few square meters in view of the coast. Contrary to other *Riotintobolus* species, such as *R. mandenensis* and *R. minutus*, *R. lavanono* sp. nov. did not remain stiff like a stick when disturbed, but rolled-up into a spiral.

#### Riotintobolus bovinus sp. nov.

Taxon classificationAnimaliaSpirobolidaPachybolidae

83B2F008-3527-5C09-B40D-B6A7C4FB2679

http://zoobank.org/5292954C-AE5F-486B-BEDE-941ADA580C1C

Figure 8

##### Material examined.

1 ♂ ***holotype***, **ZFMK MYR940**, Madagascar, Province de Toliara, Makay Mts., forêt de galerie, 21°13'27.5"S, 045°19'35.4"E, 531 m, coll. Jean Noel, 30.xi.2010.

##### Etymology:

bovinus, after the gonopods which resemble the horns of a cow. Noun in apposition.

##### Diagnosis.

*Riotintobolus bovinus* sp. nov. shares the absence of a projecting epiproct on the telson only with *R. anomalus*, *R. antafoky* sp. nov., *R. tsimelahy* sp. nov., *R. mangatsiaka* sp. nov. and *R. lavanono* sp. nov. The posterior telopod featuring two slender, sharp projections is only shared with *R. tsimelahy* sp. nov., *R. mangatsiaka* sp. nov. and *R. lavanono* sp. nov. *R. bovinus* sp. nov. differs from *R. tsimelahy* sp. nov., *R. mangatsiaka* sp. nov. and *R. lavanono* sp. nov. in a much smaller segment number and size, and strong differences in the posterior telopod, whose telopodite is uniquely shaped with two sharp processes running parallel to one another resembling a bull’s horn. *R. bovinus* sp. nov. differs by more than 14% uncorrected p-distance in the COI barcoding gene from all other *Riotintobolus* species.

##### Description.

***Measurements***: 41+0 segments. Ca. 25 mm long (broken), 2.4 mm wide.

***Colour*** (after 10 years in ethanol): Head and body rings grey, appendages red. Ventral site reddish. Posterior margins of body segments and whole margin of collum black. Anal valves black.

**Figure 8.**
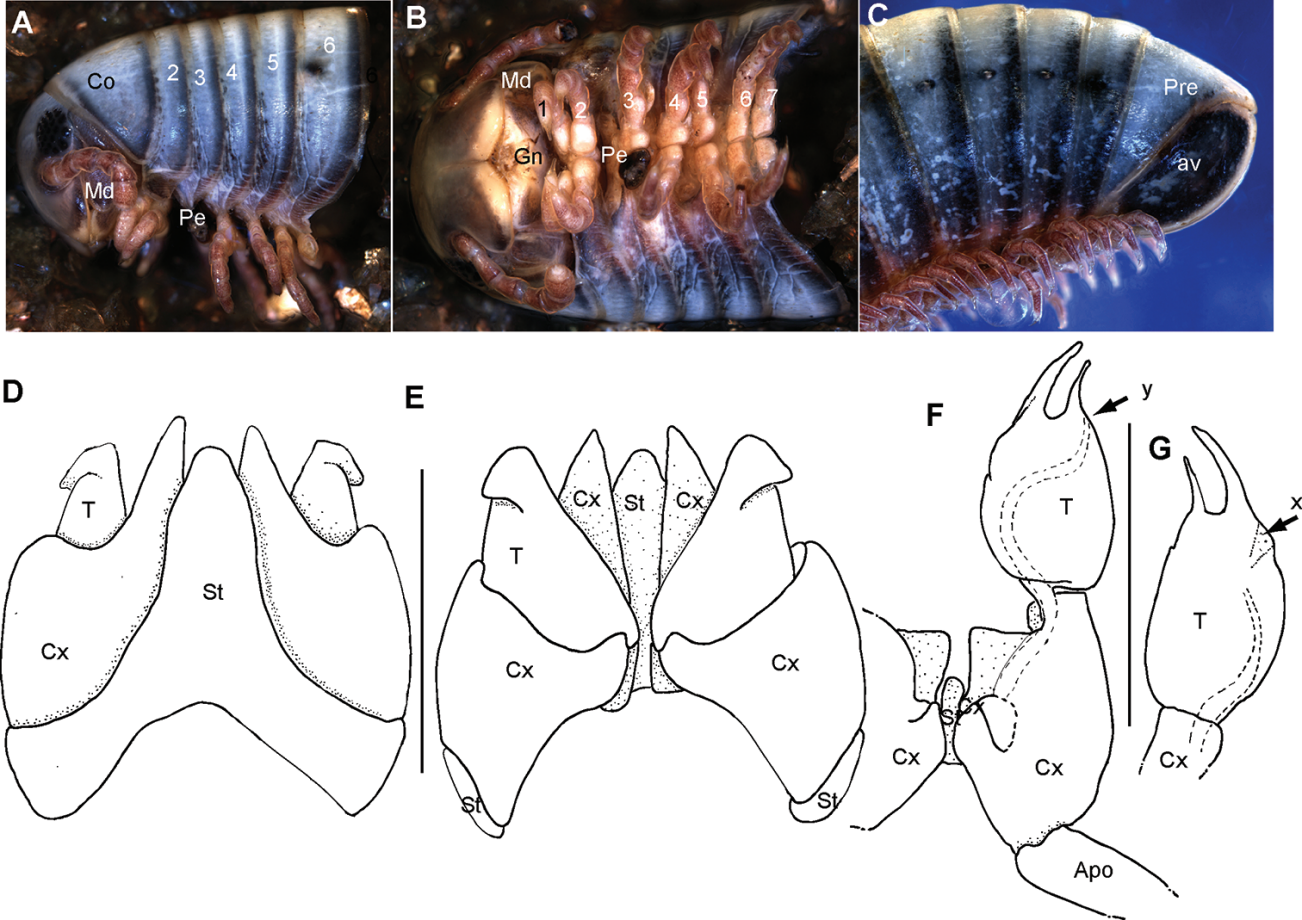
*Riotintobolus bovinus* sp. nov., male holotype (ZFMK MYR940) **A** multi-layer photograph, anterior body, lateral view **B** multi-layer photograph, leg pairs 1–7, ventral view **C** multi-layer photograph, posterior body end with telson **D** anterior gonopod, anterior view **E** anterior gonopod, posterior view **F** left posterior gonopod, posterior view **G** left posterior gonopod, anterior view. **Abbreviations**: Apo = apodeme; av = paraprocts; Co = collum (ring 1); Cx = coxite; Gn = gnathochilarium; Md = basal joints of mandible; Pe = penis; Pre = epiproct; St = sternite; T = telopodite; x = lateral swollen process; y = opening of efferenct duct; numbers refer to leg pair or segment number. Scale bars: 1 mm.

***Head***: each eye with 24–27 ommatidia in six rows. Incisura lateralis open (Fig. 8A). Labrum with standard three irregular teeth and a single row of 10–12 stout marginal setae. Clypeus with two setiferous foveolae on each side (Fig. 8B). Antennae short, protruding back to segment 3. Length of antennomeres: 1<2>3>4=5=6, second only slightly longer than 3^rd^. Terminal antennomere with four large sensory cones located together inside a membranous area (Fig. 8B). Antennomere 5 and 6 latero-apically with sensilla basiconica.

***Gnathochilarium***: lamellae linguales each with two standard setae located behind one another. Stipites each with three apical setae. Endochilarium not dissected.

***Mandible***: Stipes without projection, well rounded (FIg. 8A, B). Gnathal lobe not dissected.

***Collum***: smooth, laterally not protruding as far as ring 2 (Fig. 8A).

***Body rings***: ozopores starting at segment 6, marked by a black spot. Located slightly before, but touching suture between meso- and metazonite. Rings with smooth, but irregular coriaceous surface, ventrally on metazona with transverse ridges.

***Telson***: paraprocts without lips, abundant micropunctation especially towards edges (Fig. 8C). Epiproct well-rounded, covering, but not reaching above paraproct (Fig. 8C). Hypoproct inconspicuous (Fig. 8C).

***Legs***: leg 1 with a large cylindrical coxa, twice as long as other podomeres. Tarsus with three pairs of ventral spines and an apical spine beyond claw. Leg 2 with an elongated coxa and a strongly swollen prefemur. Tarsus with two pairs of ventral spines and a short apical spine. Midbody legs with a rectangular coxa, as long as other podomeres. Each podomere ventrally with a single or a pair of apical setae, tarsus with a tarsal pad, a single apical and two pairs of ventral spines. Length of midbody legs ca. 1.2 times body diameter in males.

***Female***: unknown.

***Male sexual characters***: tarsal pads absent (Fig. 8B). Coxae 3–7 without coxal processes (Fig. 8B).

***Anterior gonopod*** sternite massive (Fig. 8D), elongated into a wide, well-rounded triangular lobe (Fig. 8D). Sternite in anterior view well-visible, without discernible apodemes, protruding almost as high as coxal processes. Coxite with sharp triangular mesal process (Fig. 8D, E). Telopodite with slender process arising mesally (Fig. 8E), process apically curved with a short triangular projection (Fig. 8D), tip well-rounded tip, slightly protruding above lateral margin of telopodite (Fig. 8E).

***Posterior gonopods*** consisting of two parts, separated by an articulation (Fig. 8F): a long coxite and a slightly shorter telopodite, efferent duct discharging apically (Fig. 8F, G). Process of coxite and telopodite standing in same axis (Fig. 8F). Pair of posterior gonopods located parallel to each other, connected by a small, sclerotised and visible sternite (Fig. 8F). Basal part of coxite wide, mesally with a large triangular sclerite located on lower level than remaining part (Fig. 8F). Coxite elongated. Efferent duct running at mesal margin of coxite (Fig. 8F, G). Telopodite as wide as but slightly shorter than coxite, standing in same axis (Fig. 8F, G), apically membranous, with two slender apical processes resembling a bull’s horns (Fig. 8F, G). Mesal process wider and longer than lateral process. Efferent duct seems to be ending at base of mesal process (Fig. 8F, G).

##### Remarks.

*Riotintobolus bovinus* sp. nov. lives in direct sympatry with another species of the genus, *Riotintobolus makayi* sp. nov. (Fig. 2).

#### Riotintobolus antafoky sp. nov.

Taxon classificationAnimaliaSpirobolidaPachybolidae

15E9BAD2-0350-5077-8C6E-120CB922AB5C

http://zoobank.org/9F3E2163-ACA9-4A1F-B6D0-D24CD3A692D8

Figure 9

##### Material examined.

***Holotype***: ♂, **CASENT 9032794**, MGF007, Madagascar, Province Toliara, Antafoky, 80 m, spiny thicket, 23°29'16"S, 44°4'39"E, coll. Frontier project, millipede dig (3 m x 3 m), 14.xi.2001.

***Paratypes***: 1 ♂, 1 ♀, **CASENT 9032794**, same data as holotype

##### Etymology.

Antafoky, after the type locality, spiny forest of Antafoky (Fig. 2). Noun in apposition.

##### Diagnosis.

*R. antafoky* sp. nov. shares the flag-like membranous tip of the posterior gonopod with *R. mandenensis*, *R. minutus*, *R. aridus*, *R. anomalus* and *R. makayi* sp. nov. *R. antafoky* sp. nov. shares the absence of tarsal pads only with *R. bovinus* sp. nov., and the relatively simple tip of the posterior gonopod only with *R. anomalus*. *R. antafoky* sp. nov. differs from the sympatric *R. anomalus* in details of the posterior gonopod, the absence of a dorsal red stripe, the much longer antenna (protruding back to body ring 5), and the much smaller size (*R. anomalus* males 45 mm long, 4.3 mm wide, *R. antafoky* sp. nov. ca. 33 mm long, 3.2 mm wide).

##### Description.

***Measurements*** (holotype): 51+0 segments, ca. 33 mm long (fragmented) and 3.2 mm wide.

***Coloration***: segments grey, with a dark grey posterior margin, ozopore highlighted by a black spot. Head, antennae and legs dark grey.

***Head***: each eye with 34 ommatidia in six rows. Incisura lateralis open. Labrum with standard three irregular teeth and a single row of 10–12 stout marginal setae. Clypeus with two setiferous foveolae on each side. Antennae long, protruding back to segment 5. Terminal antennomere with four large sensory cones located together inside a membranous area. Antennomere 5 and 6 latero-apically with sensilla basiconica.

***Gnathochilarium***: lamellae linguales each with two standard setae located behind one another. Stipites each with three apical setae. Endochilarium not dissected.

***Mandible***: Stipes without projection, well rounded. Gnathal lobe not dissected.

***Collum***: smooth, laterally not protruding as far as ring 2.

***Body rings***: ozopores starting at segment 6, marked by a black spot. Located slightly before, but touching suture between meso- and metazonite. Rings with smooth, but irregular coriaceous surface, ventrally on metazona with transverse ridges.

***Telson***: paraprocts without lips, abundant micropunctation especially towards edges. Epiproct well-rounded, covering, but not reaching above paraproct. Hypoproct inconspicuous.

***Legs***: leg 1 with a large cylindrical coxa, twice as long as other podomeres. Tarsus with three pairs of ventral spines and an apical spine beyond claw. Leg 2 with an elongated coxa and a strongly swollen prefemur. Tarsus with two pairs of ventral spines and a short apical spine. Midbody legs with a rectangular coxa, as long as other podomeres. Each podomere ventrally with a single or a pair of apical setae, tarsus without a tarsal pad, a single apical and two pairs of ventral spines. Length of midbody legs ca. 0.9 times body diameter in males.

***Female***: not investigated.

***Male sexual characters***: tarsal pads absent. Coxae 3–7 without coxal processes, but coxae 6 and 7 flattened, rectangular.

**Figure 9.**
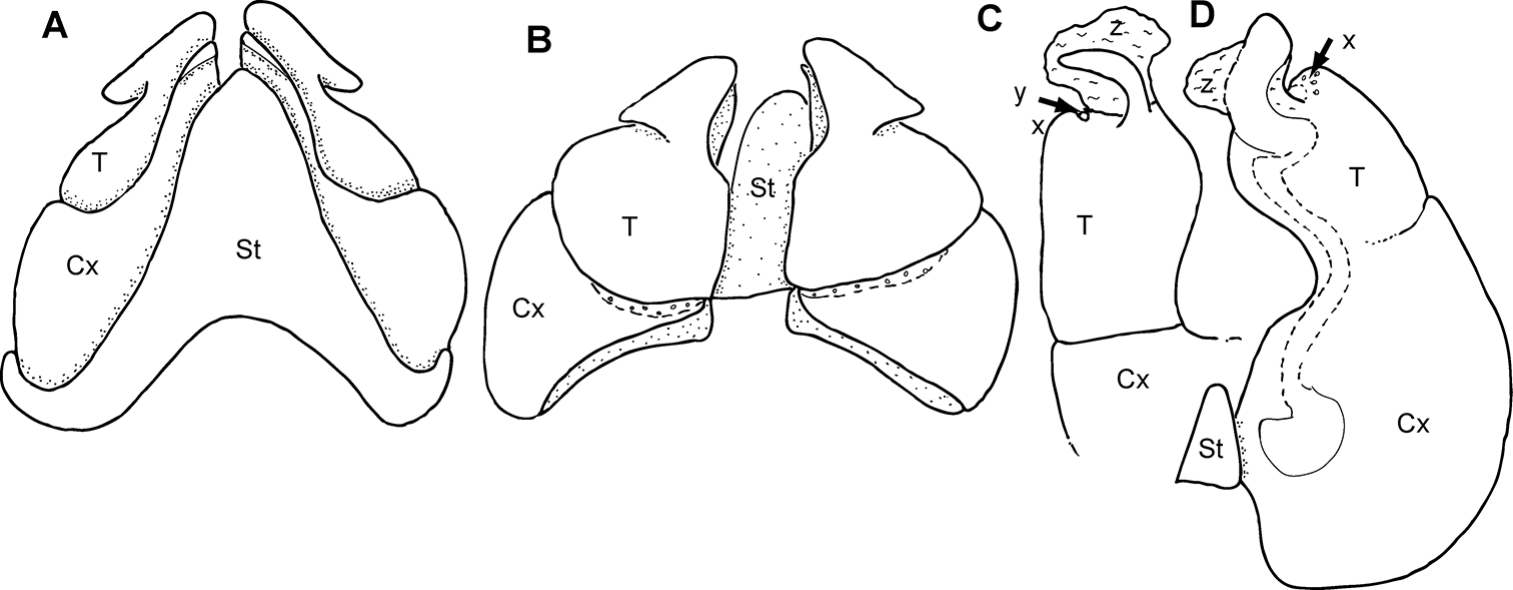
*Riotintobolus antafoky* sp. nov., holotype (CASENT9032794) **A** anterior gonopod, anterior view **B** anterior gonopod, posterior view **C** telopodite of left posterior gonopod, anterior view **D** left posterior gonopod, posterior view. **Abbreviations**: Cx = coxite; St = sternite; T = telopodite; x = lateral swollen process; y = opening of efferenct duct; z = apical membranous ‘flag’. Not to scale.

***Anterior gonopod*** sternite massive (Fig. 9A), elongated into a wide, well-rounded triangular lobe (Fig. 9A). Sternite in anterior view well-visible, without discernible apodemes, protruding almost as high as coxal processes. Coxite with sharp triangular mesal process (Fig. 9A, B), upper rim bending forward in anterior view (Fig. 9A). Telopodite with strong process arising mesally (Fig. 9B), process apically curved with a short triangular projection (Fig. 9A), tip well-rounded, protruding above lateral margin of telopodite (Fig. 9B).

***Posterior gonopods*** consisting of two parts, separated by an articulation: a long coxite and a slightly shorter telopodite, efferent duct discharging apically (Fig. 9C, D). Process of coxite and telopodite standing in same axis (Fig. 9D). Pair of posterior gonopods located parallel to each other, connected by a small, sclerotised and visible sternite (Fig. 9D). Basal part of coxite wide, mesally with a triangular sclerite located on lower level than remaining part (Fig. 9C). Coxite elongated. Efferent duct running at mesal margin of coxite (Fig. 9C, D). Telopodite as wide as but slightly shorter than coxite, standing in same axis (Fig. 9D), apically with a membranous ‘flag’ (Fig. 9C, D). Laterally with sclerotised projection bending laterally and completely surrounded by membranous flag. (Fig. 9C, D). Efferent duct ending at base of process (Fig. 9C, D). Apex laterally with weakly developed finger-shaped process.

##### Remarks.

this species lives in direct sympatry with the much larger *R. anomalus*.

#### Riotintobolus makayi sp. nov.

Taxon classificationAnimaliaSpirobolidaPachybolidae

351673C0-372A-5B05-9A31-0174A6FBC82D

http://zoobank.org/233B0416-1281-4AB0-8D12-72688CFEE696

Figure 10

##### Material examined.

1 ♂ ***holotype***, **ZFMK MYR7173**, Madagascar, Province de Toliara, Makay Mts., forêt de galerie, 21°13'25.1"S, 045°19'36.0"E, 512 m, coll. Jean Noel, 29.xi.2010. Paratypes: 1 ♂, 1 ♀, **ZFMK MYR939**, Madagascar, Province de Toliara, Makay Mts., forêt de galerie, 21°13'27.5"S, 045°19'35.4"E, 531 m, coll. Jean Noel, 01.xii.2010.

##### Etymology.

Makayi, after the type locality, the area of Makay (Fig. 2). Noun in apposition.

##### Diagnosis.

*R. makayi* sp. nov. shares the flag-like membranous tip of the posterior gonopod with *R. mandenensis*, *R. minutus*, *R. aridus*, *R. anomalus* and *R. antafoky* sp. nov. *Riotintobolus makayi* sp. nov. shares the wide dorsal stripe, presence of tarsal pads on at least male legs 3–7, and a projecting epiproct only with *R. mandenensis*, *R. minutus*, and *R. aridus*. *R. makayi* sp. nov. differs from *R. mandenensis* and *R. minutus* in the configuration of the flag of the posterior telopod, which consists only of a single fold, while it has two folds in the latter two species. The curved lateral process and the freely ending efferent duct are unique characters for *R. makayi* sp. nov.

**Description** (based on male holotype): ***Measurements***: Male holotype: 38+0 segments. Ca. 22 mm long (broken), 2.4 mm wide. Female paratype: 38+0 segments. Ca. 33 mm long (broken), 3.5 mm wide.

**Figure 10.**
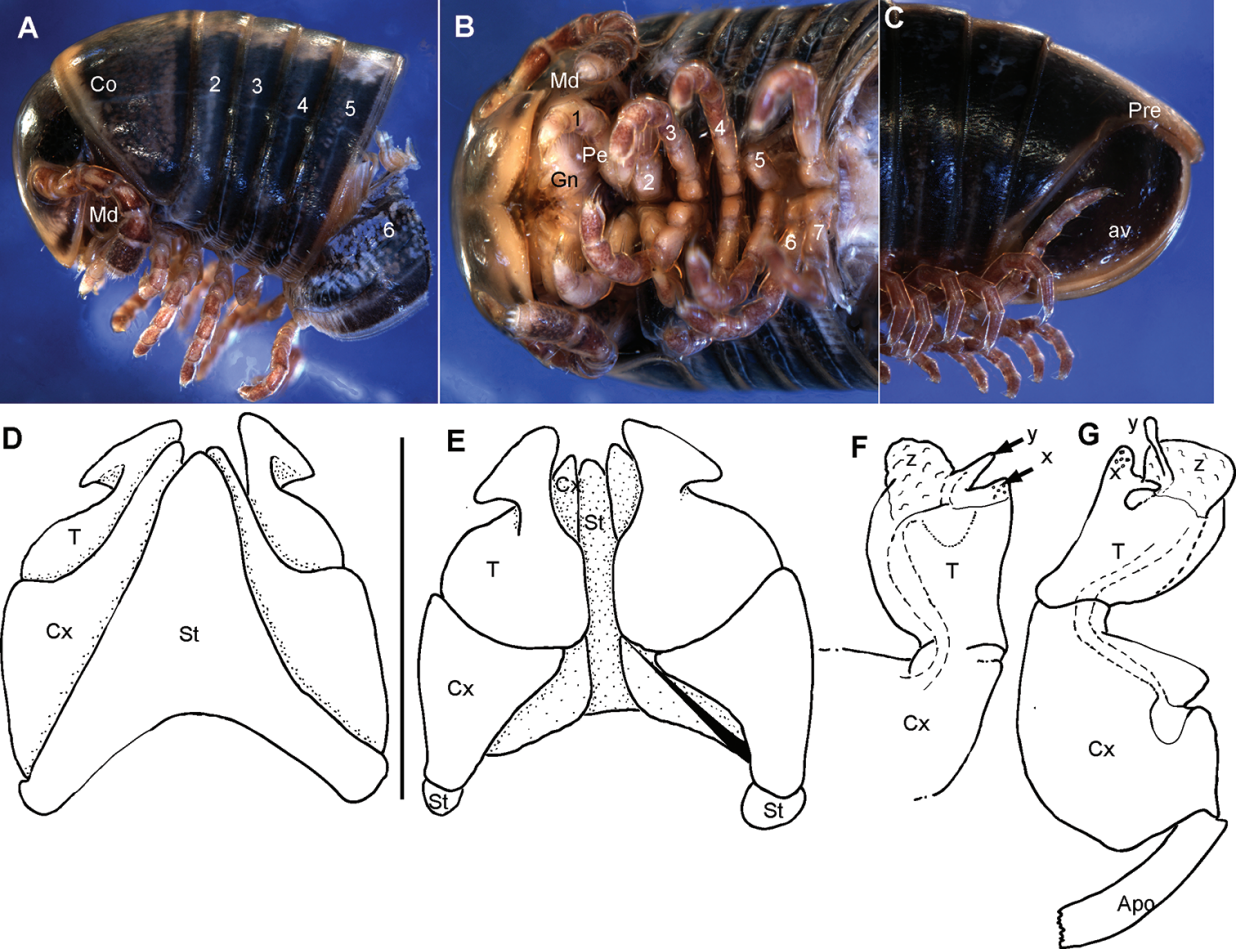
*Riotintobolus makayi* sp. nov., male holotype (ZFMK MYR939) **A** multi-layer photograph, anterior body, lateral view **B** multi-layer photograph, leg pairs 1–7, ventral view **C** multi-layer photograph, posterior body end with telson **D** anterior gonopod, anterior view **E** anterior gonopod, posterior view **F** right posterior gonopod, anterior view **G** right posterior gonopod, posterior view. **Abbreviations**: Apo = apodeme; av = paraprocts; Co = collum (ring 1); Cx = coxite; Gn = gnathochilarium; Md = basal joints of mandible; Pre = epiproct; St = sternite; T = telopodite; x = lateral swollen process; y = opening of efferenct duct; z = apical membranous ‘flag’; numbers refer to leg pair or segment number. Scale bars: 1 mm.

***Colour*** (after ten years in ethanol): Head except for light clypeus black, body rings black, appendages red (Fig. 10A, B). Anterior margin of collum light brown. Dorsally with two wide light brown-reddish stripes, divided by a black stripe, all stripes even crossing the epiproct. Anal valves black, margin light brown, hypoproct light brown (Fig. 10C).

***Head***: each eye with 26–28 ommatidia in six rows. Incisura lateralis open (Fig. 10A). Labrum with standard three irregular teeth and a single row of 10–12 stout marginal setae. Clypeus with two setiferous foveolae on each side (Fig. 10B). Antennae short, protruding back to segment 3. Length of antennomeres: 1<2=3=4=5=6. Terminal antennomere with four large sensory cones located together inside a membranous area (Fig. 10B). Antennomere 5 and 6 latero-apically with sensilla basiconica.

***Gnathochilarium***: lamellae linguales each with two standard setae located behind one another. Stipites each with three apical setae. Endochilarium not dissected.

***Mandible***: Stipes without projection, well rounded (Fig. 10A, B). Gnathal lobe not dissected.

***Collum***: smooth, laterally not protruding as far as ring 2 (Fig. 10A).

***Body rings***: ozopores starting at segment 6, located slightly before, but touching suture between meso- and metazonite. Rings with smooth, but irregular coriaceous surface, ventrally on metazona with transverse ridges.

***Telson***: paraprocts with lips, abundant micropunctation especially towards edges (Fig. 10C). Epiproct well-rounded, reaching slightly above paraproct with a massive process (Fig. 10C). Hypoproct inconspicuous (Fig. 10C).

***Legs***: leg 1 with a large cylindrical coxa, twice as long as other podomeres. Tarsus with three pairs of ventral spines and an apical spine beyond claw. Leg 2 with an elongated coxa. Tarsus with two pairs of ventral spines and a short apical spine. Midbody legs with a rectangular coxa, as long as other podomeres. Each podomere ventrally with a single or a pair of apical setae, tarsus of leg 3– midbody with a tarsal pad, a single apical and three or four pairs of ventral spines (Fig. 10B). Legs on posterior part of body without a tarsal pad and two or three pairs of ventral spines. Length of midbody legs ca. 1.2 times body diameter in male.

***Female sexual characters*.** No tarsal pads, antennae even shorter than male, only protruding back to collum. Female vulva simple, bivalve-like.

***Male sexual characters***: tarsal pads present from leg 3 to midbody (Fig. 10B). Coxae 3–7 without coxal processes (Fig. 10B).

***Anterior gonopod*** sternite massive, elongated into a wide, well-rounded, triangular lobe (Fig. 10D). Sternite in anterior view well-visible, without discernible apodemes, protruding almost as high as coxal processes. Coxite with sharp triangular mesal process (Fig. 10D, E). Telopodite with slender process arising mesally (Fig. 10E), process apically curved with a strong triangular projection (Fig. 10D), tip well-rounded tip, protruding above lateral margin of telopodite (Fig. 10C).

***Posterior gonopods*** consisting of two parts, separated by an articulation: a long coxite and a slightly shorter telopodite, efferent duct discharging apically (Fig. 10G). Process of coxite and telopodite standing in same axis (Fig. 10F, G). Pair of posterior gonopods located parallel to each other, connected by a small, sclerotised and visible sternite (Fig. 10G). Basal part of coxite wide, mesally with a triangular sclerite located on lower level than remaining part (Fig. 10G). Coxite elongated. Efferent duct running at mesal margin of coxite (Fig. 10F, G). Telopodite as wide as but slightly shorter than coxite, standing in same axis, apically with a membranous ‘flag’ (Fig. 10F, G). Laterally with sclerotised projection with a slender mesad-orientated branch (Fig. 10F, G). Membranous ‘flag’ longer than lateral process. Efferent duct ending freely, projecting above ‘flag apically (Fig. 10F, G).

##### Comments.

this species lives in direct sympatry with *Riotintobolus bovinus* sp. nov. The female carried eggs in the body.

## Discussion

### Genetic distances in the Barcoding gene of *Riotintobolus* species compared to other Diplopoda

The interspecific distance of 11–16.4% between *Riotintobolus* species is comparable to those found in the only other Malagasy Spirobolida genus for which molecular data are available, *Aphistogoniulus*. *Aphistogoniulus* species are much more widespread and showing even higher interspecific distances (Wesener et al. 2009b, 2011a), being found all over Madagascar, while *Riotintobolus* is currently known only from the South. For other millipedes from Madagascar, interspecific distances are known for two genera of giant pill-millipedes (Sphaerotheriida), *Sphaeromimus* DeSaussure & Zehntner, 1902 and *Zoosphaerium* Pocock, 1895. In *Sphaeromimus*, whose species show a distribution restricted to southern Madagascar comparable to those of *Riotintobolus*, interspecific distances vary mainly between 8.3–20.8% (Wesener et al. 2014; Moritz and Wesener 2017). In the widespread genus *Zoosphaerium*, only a handful of species were sequenced (Wesener et al. 2010; Sagorny and Wesener 2017; Wesener and Anilkumar 2020), with interspecific distances varying between 9.1–16.3%.

### A hidden soil arthropod diversity in Madagascar’s driest areas

Our discovery of six new species, especially of the occurrence of two unrelated species of *Riotintobolus* in direct sympatry and the presence of an additional candidate species, shows that *Riotintobolus* is an important component of the soil macrofauna in Madagascar’s spiny forest ecosystem.

## Supplementary Material

XML Treatment for Riotintobolus

XML Treatment for Riotintobolus tsimelahy

XML Treatment for Riotintobolus mangatsiaka

XML Treatment for Riotintobolus lavanono

XML Treatment for Riotintobolus bovinus

XML Treatment for Riotintobolus antafoky

XML Treatment for Riotintobolus makayi

## Supplementary materials

Supplementary material 1 P-Distances between Riotintobolus species Data type p-distances Explanation note The number of base differences per site from between sequences are shown. The analysis involved 19 nucleotide sequences. Codon positions included were 1st+2nd+3rd. All ambiguous positions were removed for each sequence pair. https://binary.pensoft.net/file/436172This dataset is made available under the Open Database License (http://opendatacommons.org/licenses/odbl/1.0/). The Open Database License (ODbL) is a license agreement intended to allow users to freely share, modify, and use this Dataset while maintaining this same freedom for others, provided that the original source and author(s) are credited.Author: Thomas Wesener
